# Pressure induced mechanical, elastic, and optoelectronic characteristics of Cd_0.75_Zn_0.25_Se alloy

**DOI:** 10.3389/fchem.2024.1405315

**Published:** 2024-08-01

**Authors:** Muhammad Aamir Iqbal, Saher Javeed, Sunila Bakhsh, Iván D. Arellano-Ramírez, Muhammad Khalid, Kareem Morsy, Ali A. Shati, Jeong Ryeol Choi

**Affiliations:** ^1^ School of Materials Science and Engineering, Zhejiang University, Hangzhou, China; ^2^ Department of Physics, Government College University Lahore, Lahore, Pakistan; ^3^ Department of Physics, Balochistan University of Information Technology, Engineering and Management Sciences, Quetta, Pakistan; ^4^ Department of Physics, Universidad Tecnológica de Pereira, Pereira, Colombia; ^5^ Biology Department, College of Science, King Khalid University, Abha, Saudi Arabia; ^6^ School of Electronic Engineering, Kyonggi University, Suwon, Republic of Korea

**Keywords:** Cd_0.75_Zn_0.25_Se alloy, density functional theory, pressure, bandgap, visible display, optical properties

## Abstract

The change in composition and pressure, both of which lead to new desired properties by altering the structure, is particularly important for improving device performance. Given this, we focused here on the mechanical, elastic, and optoelectronic characteristics of the Cd_0.75_Zn_0.25_Se alloy using density functional theory at various pressures from 0 GPa to 20 GPa. It is found that the bulk modulus of the material rises with increasing pressure and exhibits mechanical stability as well as cubic symmetry. In addition, the increased pressure leads to a rise in the direct bandgap energy of the material from 2.03 eV to 2.48 eV. The absorption coefficient of the alloy also increases as the pressure increases, where the effective range of absorption covers the broad spectrum of light in the visible range from orange to cyan. This is due to the electronic transitions caused by the altered pressure. The optical parameters, including optical conductivity, extinction coefficient, reflection, and refractive index, are also analyzed under the influence of pressure. Based on this research, effective applications of the Cd substituted Zn-chalcogenides (CdZnSe) alloys in the fields of optoelectronics and photovoltaics are outlined, especially concerning fabricating solar cells, photonic devices, and pressure sensors for space technology.

## 1 Introduction

Technological advances have enabled us to overcome the commercial challenges of achieving the desired properties of ternary alloys by adjusting their composition. For instance, it is particularly interesting to tune the characteristics of the semiconductors from the II to VI group by doping and regulating thermodynamical parameters such as pressure with the scheme of broadening the light absorption spectrum and making them available in high-pressure optoelectronic devices (Krishnan et al., 2019; Gul et al., 2022). Under these modifications, the bandgap of the material can be tailored to its direct bandgap nature, which plays a key role when utilized in photovoltaic and optoelectronic industries where the material is exposed to pressure and compositional influence. Such bandgap modifications help to frame various types of devices such as photodetectors, solar cells, sensors, light-emitting diodes (LEDs), and even other photovoltaic and optoelectronic devices covering a broad spectral range (Benkabou et al., 2000; Mahmood et al., 2017; Iqbal et al., 2022a; Liu et al., 2022; Iqbal et al., 2023a; Iqbal et al., 2023b).

Conventionally, it is shown that the CdZnSe alloys are suitable for photoconductive and photoluminescent devices owing to their high stability and available ranges of absorption spectrum (Sutrave et al., 2000; Zhang et al., 2017; Zhang et al., 2019; Nguyen et al., 2022). Previous studies have reported various synthesis methods for CdZnSe thin films, such as molecular beam epitaxy, chemical bath deposition, vacuum evaporation, and electrodeposition, together with their structural, electronic, magnetic, and dielectric properties (Gupta et al., 1995; Loglio et al., 2008; Murali and Austine, 2009; Margapoti et al., 2012; Deo et al., 2014; Jin et al., 2021; Loghina et al., 2021). Loghina *et al.* reported an experimental method for synthesizing CdZnSe quantum dots with a direct bandgap of about 2.27 eV (Loghina et al., 2019), while a CASTEP-applied study based on a plane wave pseudopotential method reported the optical and electronic properties of the same alloy (Korozlu et al., 2011). In another study, the thermodynamic properties of the CdZnSe alloys were investigated using Quantum Expresso software in the temperature range from 0 K to 1000 K along with the pressure variations from 0 GPa to 10 GPa, and this was followed by investigations on their thermal conductivity (Aarifeen and Afaq, 2017; ul Aarifeen and Afaq, 2020). In addition, the structural and electronic characteristics of the same ternary alloy at room temperature have been studied using the first-principles method (Ameri et al., 2012). So far, all these methods have provided a basic understanding of the material properties, which necessitates that we identify the deficiencies and address them further.

As can be seen from the available literature and previous studies, the investigation of optical properties of the alloy Cd_0.75_Zn_0.25_Se under high pressure is not reported, whereas there are few studies investigating its significance both experimentally and theoretically. To analyze the pressure-influenced characteristics, this study aims to investigate the physical phenomena of this CdZnSe semiconductor alloy under pressure fluctuations and further explore their optoelectronic and pressure sensor applications in industry. The structural characteristics of this ternary alloy at high pressure were analyzed in terms of its stability, for the first time, using the generalized gradient approximation (GGA) functional based on density functional theory (DFT). In particular, the elasto-mechanical and optoelectronic characteristics of the Cd_0.75_Zn_0.25_Se are investigated using GGA and modified Becke-Jhonson (mBJ) potential, respectively. This specific alloy composition consisting of 75% Cd and 25% Zn is critical to the improvement of optical behavior via changing pressure, as its effective light absorption takes place only in the visible range from orange to cyan, which can be used in display applications including LEDs and solar cell coatings. This study is significant because, from the outset, it may serve as a platform for pressure-driven research in a wide range of optoelectronics and photonic fields.

## 2 Simulation method

The computational investigations for solving the Kohn–Sham equations were completed with the framework of DFT (Iqbal et al., 2021) using the computer software Wien2k (Blaha et al., 2001). This technique has been proven to be popular and important for theoretical calculations by illustrating the numerous results of its application. In this framework, the energy separation of core and valence energy states was set at −6.0 Ry, while the core had a high spherical harmonic potential up to the value of *l*_max_ = 10, where *l*_max_ is the highest value of the harmonic order *l*. The force, energy, and charge convergence parameters were set to one mRy/Bohr, 0.00001 Ry, and 0.00001 e, respectively, whereas the value R_MT_×K_max_ was set to 7.0, while R_MT_ denotes the smallest of the muffin-tin sphere radii and K_max_ is the largest reciprocal lattice vector. However, the muffin-tin sphere radius (RMT) values for Cd and Zn were set at 2.37 Bohr, but for Se at 2.26 Bohr. The grid for k-point sampling was located in the irreducible Brillouin zone with an order of 12 × 12 × 12. The structures were optimized using GGA-PBEsol and by generating a supercell of 1 × 1 × 1 dimension in which the 75% Zn atoms were replaced with Cd atoms to generate the pressure-dependent structures of the Cd_0.75_Zn_0.25_Se alloy. The optimized lattice constants obtained at varying pressures were further employed to introduce the pressure impact in the range of 0–20 GPa. To analyze the elastic and mechanical characteristics, we employed GGA functional, while mBJ potential (Singh, 2010) was used to compute optoelectronic properties. The electronic bandgaps were also computed using the EV-GGA functional, established by Engel and Vosko as a modification of the GGA functional that computes the bandgap and exchange potential with higher productivity (Engel and Vosko, 1993).

The main objective of this study is to find the pressure-induced band structures and tunable optical properties by ensuring their stability under the effect of altering pressure, which can be achieved by adjusting the stability of the ternary alloy. In materials physics, structural parameters are crucial, as they enable data collection on the microscopic structure of materials and greatly impact the forecast of further properties. The cubic phase structural parameters of the alloy, including the lattice constant, were calculated by the pressure effects using the equation (Sahli et al., 2016) ${a\left(P\right)=a}_{0}{\left[1+P\frac{B^{\prime }}{B_{0}}\right]}^{\frac{-1}{{3B}^{\prime }}}$ , where a_0_ is the lattice constant at 0 GPa pressure, B_0_ the bulk modulus, and B^**/**^ the pressure derivative of the bulk modulus. Extensive studies along this line have enabled clarification of pressure-induced changes in the lattice parameters and their effects on materials’ physical properties from the theoretical point of view based on this equation. The obtained results agreed well with experimental results, which provides us with the reliability of using this equation to study the effects of pressure on the band structures and optical characteristics of the Cd_0.75_Zn_0.25_Se alloy.

## 3 Results and discussion

This section is dedicated to discussing the structural stability by validating the Born stability criterion of this ternary alloy and its optoelectronic properties to analyze the electronic mechanism that is responsible for tuning the performance of the device. We investigated the potential of its use in electronic and optical devices to obtain a specific color display at changing pressure associated with the change in depth or altitude.

### 3.1 Elastic properties, structural and mechanical stability

**FIGURE 1:**
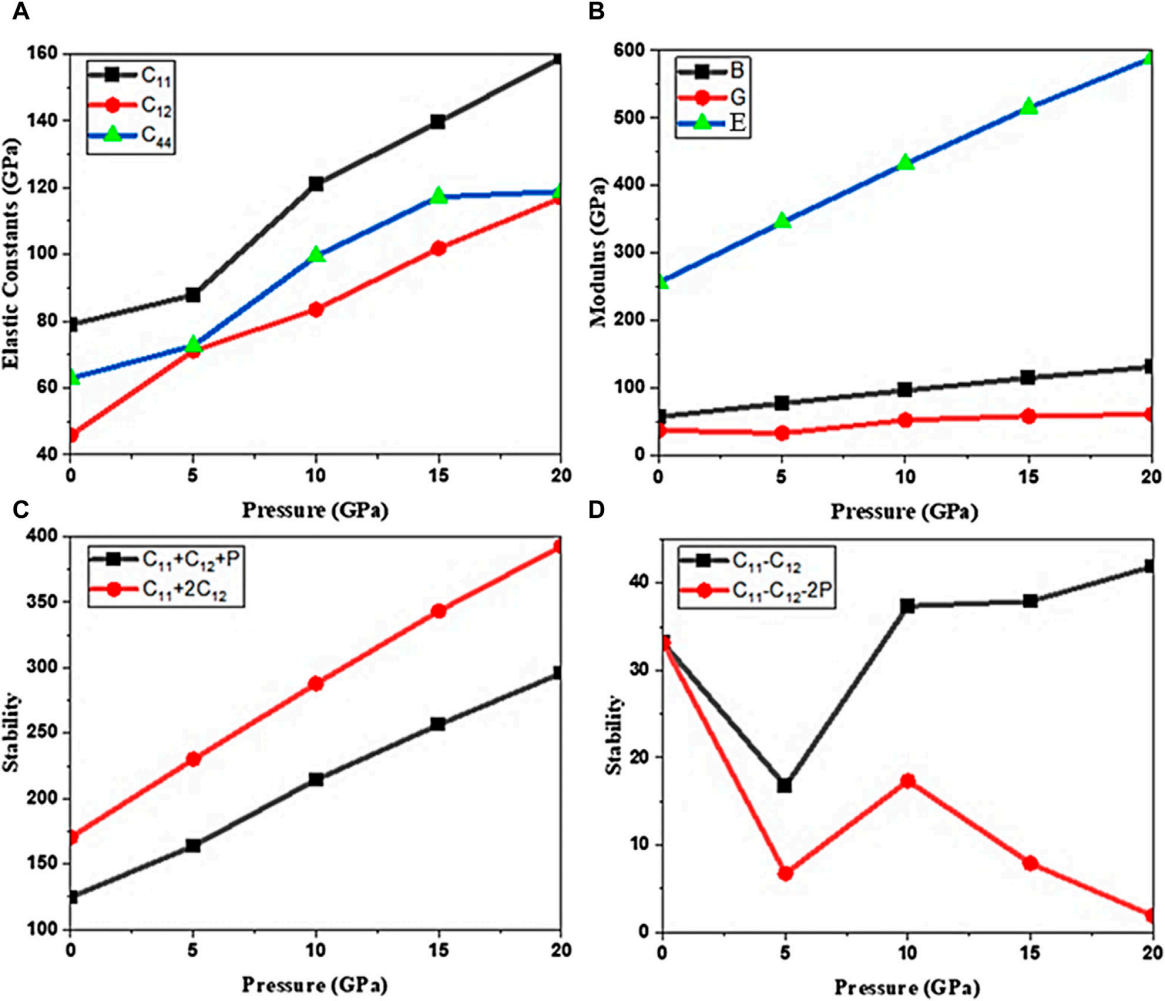
Moduli of elasticity under the impact of pressure. **(A)** Cubic elastic constants, **(B)** cubic elastic moduli, **(C)** stability criteria C_11_+2C_12_ > 0 and C_11_+2C_12_ + P > 0, and **(D)** stability conditions C_11_-C_12_ > 0 and C_11_-C_12_-2P > 0.

The Cd_0.75_Zn_0.25_Se alloy exhibits a cubic zinc blende phase at 0 GPa pressure, as shown in the Supplementary Material (see Supplementary Figure S1). We computed the structural characteristics of the alloy to further approximate the elastic constants to analyze the elastic and mechanical stability of the material at different pressures. Pressure changes the lattice parameters that were further used to explore stability by investigating the behavior of elastic constants. The computed outcomes show that as the pressure rises, the lattice constant decreases (see Supplementary Figure S2), resulting in a reduction in the volume of the unit cell. Due to the repulsive contact between atoms, the compression of the crystal is not uniform over the complete pressure range. That is, the relative compression of the structure decreases as pressure increases. Moreover, the lattice constant and cell volume decrease as pressure increases because the atoms are closer together and have a stronger repulsive connection (Mahmood et al., 2017). In contrast to this, the elastic constant values of the alloyed materials are increased. These outcomes satisfy the stability conditions of the cubic crystals with the three elastic constants C_44_, C_12,_ and C_11_, which are also used to examine the conformity of the Born stability criteria (Milstein and Hill, 1979). In addition, for pressures 0–20 GPa, the structure retains the cubical symmetry, but at higher pressures than 20 GPa, there is a phase transition and the cubical symmetry is breached. In this work, we remained restricted to studying the cubic phase’s pressure-driven characteristics. The structural parameters, along with a summary of elastic constant data and stability conditions, are presented in Supplementary Table S1. There are three conditions as the criteria for the mechanical stability of the alloy at normal room temperature: namely, C_11_-C_12_ > 0, C_44_ > 0, and C_11_+2C_12_ > 0. On the other hand, the stability criteria at high pressure are as follows: C_11_ + C_12_ + P > 0, C_11_-C_12_-2P > 0, and C_44_-P > 0. If the alloy meets these two criteria, it is considered mechanically stable in the applied pressure range. Here, these criteria are achieved and therefore this ternary alloy is stable at pressures ranging from 0 GPa to 20 GPa (see Figure 1). As pressure changes the lattice constant, the elastic constants also change significantly. At a pressure of 5 GPa, a sudden decrease in the outcomes of the stability conditions C_11_-C_12_ > 0, and C_11_-C_12_-2P > 0 can be seen. This is due to the compression involved in the presence of the applied pressure. However, cubic stability is still retained. Also, Young’s modulus value increases in proportion to the pressure. This is due to the reason that the atoms are pressed closer together, making the substance stiff. Other parameters affected by pressure variation are elastic coefficients, mechanical parameters (see Figures 1A,B), stability conditions (see Figures 1C,D), and lattice constants (see Supplementary Figure S2A). From the calculated data, the ternary alloys of group II-VI can be used efficiently in optoelectronic devices due to their mechanical stability as a cubic phase at high-pressure ranges (Aarifeen and Afaq, 2017; ul Aarifeen and Afaq, 2020; Iqbal et al., 2022b).

The mechanical stability of the Cd_0.75_Zn_0.25_Se alloy has been discussed using various parameters, such as bulk modulus (B), Young’s modulus (E), anisotropy factor (A), shear modulus (G), Cauchy pressure (CP), Poisson’s ratio (υ), and Pugh ratio (Kishore et al., 2019; Pu et al., 2019) using the Equations 1-9. The bulk modulus can be mathematically determined from:

$$B=\frac{B_{v}+B_{R}}{2}$$ (1)

where B_v_ denotes Voigt bulk modulus and B_R_ Reuss bulk modulus, which can be written as:

$$B_{v}=B_{R}=\frac{C_{11}+2C_{12}}{3}$$ (2)

The shear modulus is also used to study the stiffness of the material. The shear modulus is also given in a similar way, but in terms of Voigt shear modulus G_v_ and Reuss shear modulus G_R_ such that

$$G=\frac{G_{v}+G_{R}}{2}$$ (3)

In our case, G_V_ and G_R_ are represented as:

$$G_{V}=\frac{1}{5}\;\left(3C_{44}+C_{11}-\;C_{12}\right)$$ (4)

$$G_{R}=\frac{5\left(C_{11}-\;C_{12}\right)C_{44}}{3\left(C_{11}-\;C_{12}\right){\text{ `}+\;4C}_{44}}$$ (5)

Both the bulk modulus and shear modulus can be used together to compute Poisson’s ratio (υ) and Young’s modulus (E) of the ternary alloy, where they are given by (Kishore et al., 2019; Yang et al., 2020)

$$\upsilon =\frac{3B-G}{2\;\left(3G+B\right)}$$ (6)

$$E=\frac{9GB}{3G+B}$$ (7)

In addition, Cauchy pressure (CP) and anisotropic factor (A) can be expressed as follows:

$$\text{CP}=C_{12}-\;C_{44}$$ (8)

$$A=\frac{2C_{44}}{C_{11}-\;C_{12}}$$ (9)

To determine the brittleness or ductility of a material, the ratio of bulk to shear modulus (B/G), known as the Pugh ratio has been used. The material is considered to be ductile if the Pugh ratio is above 1.75, while otherwise it is considered brittle (Kishore et al., 2019; Pu et al., 2019; Yang et al., 2020; Iqbal et al., 2022b). In the present study, the Cd_0.75_Zn_0.25_Se alloy has a Pugh ratio greater than 1.75 at pressure variations from 5 GPa to 20 GPa, confirming its ductility when we apply such pressures (see Supplementary Figure S2C,D), while the alloy has a Pugh ratio less than 1.75 at ambient pressure (0 GPa), confirming its brittle character without pressure treatment. It can be deduced that the pressure treatment causes a shift in material nature from brittle to ductile. Similarly, CP and υ are computed to investigate the mechanical characteristics of the material, as the material is considered ductile if the value of CP’s is positive, otherwise, it is considered brittle (De Waele et al., 2016). Furthermore, the anisotropy factor of the material is considered to determine its mechanical properties, while the material is isotropic if the anisotropy factor is 1. It is considered to be anisotropic when the anisotropy factor is greater than 1. We conclude that this material is anisotropic in the pressure range of 0 GPa–20 GPa since the anisotropy factor is greater than one at all applied pressures (see Supplementary Figure S2B). Table 1 shows the quantitative properties of the Cd_0.75_Zn_0.25_Se alloy through the list of various physical factors calculated, such as shear modulus, bulk modulus, anisotropy factor, Pugh’s ratio, Poisson’s ratio, elastic modulus, and Cauchy’s pressure.

**TABLE 1:** Young’s modulus E (GPa), Voigt shear modulus G_V_ (GPa), Reuss shear modulus G_R_ (GPa), shear modulus G (GPa), anisotropy ratio A, Pugh’s ratio B/G, and G/B ratio depending on the pressure *p* (GPa) for Cd_0.75_Zn_0.25_Se alloy.

P	E	G_v_	G_R_	G	A	B/G	G/B
0	255.81	44.34	29.68	37.02	3.79	1.53	0.65
5	345.03	46.93	17.84	32.39	8.67	2.37	0.42
10	431.67	67.13	36.43	51.78	5.32	1.85	0.54
15	514.86	77.89	38.13	58.01	6.18	1.97	0.51
20	589.01	79.64	41.42	60.53	5.67	2.16	0.46

### 3.2 Electronic properties

To estimate the capability of a material for optoelectronic and photonic applications, it is necessary to know its electronic characteristics induced by electronic transitions. One can study and understand electronic behavior by estimating material properties under pressure and then extrapolating how much-concentrated alloy material is needed for a particular device application. The electronic characteristics of the Cd_0.75_Zn_0.25_Se alloy, including the density of states (DOS) and the band structures (BS), are studied at pressure variations in the Brillouin zone with high peak symmetry points. The effect of pressure variations on the BS’s was investigated using calculation functionals such as EV-GGA, GGA, and mBJ potential, while the lattice parameters were calculated using the GGA only. Using GGA, the pressure influence on the lattice constant was acquainted with and incorporated into the BS’s within mBJ and EV-GGA. The result of this study shows the direct bandgap characteristic of the Cd_0.75_Zn_0.25_Se alloy at all applied pressures.

**FIGURE 2:**
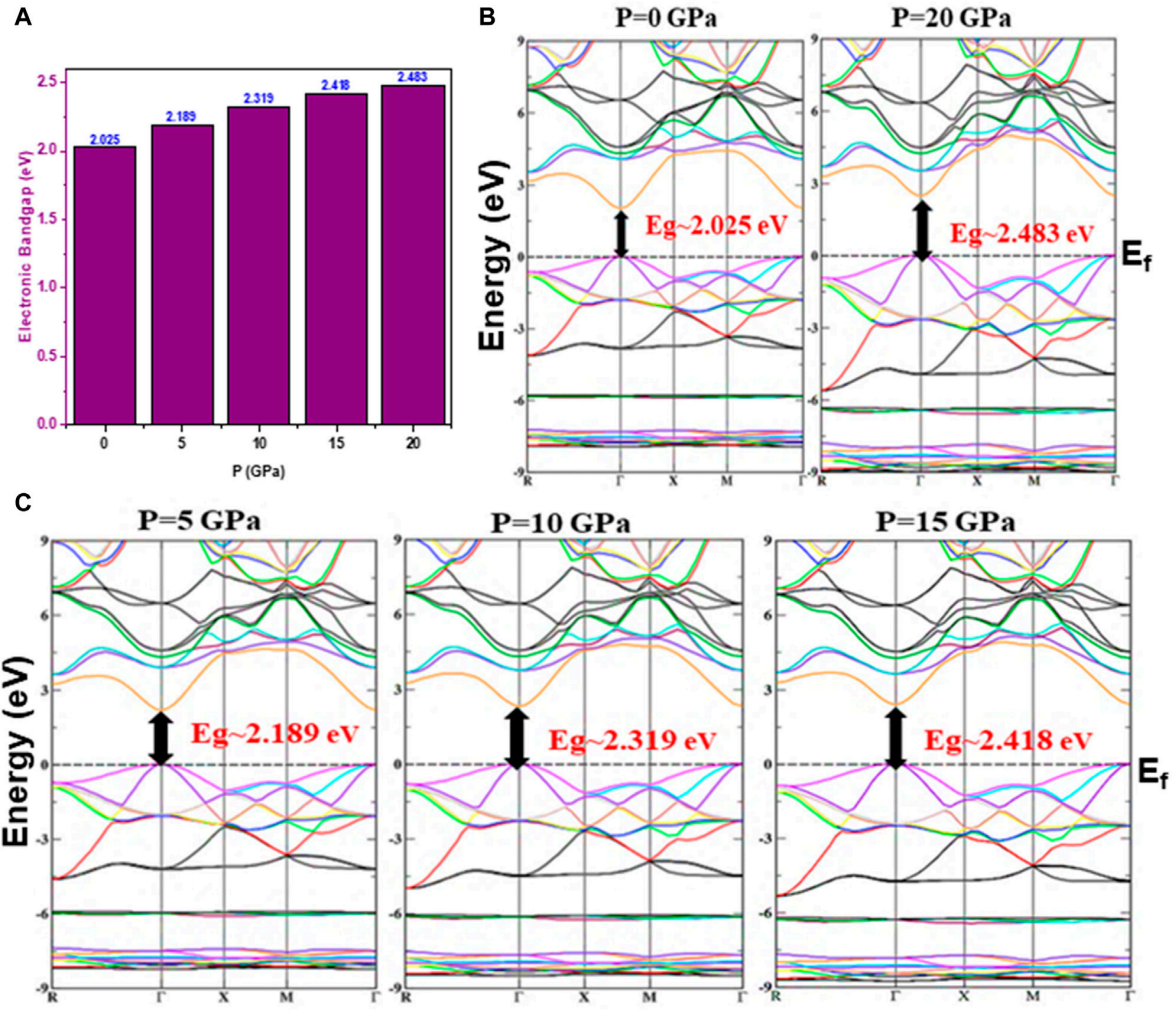
Pressure-influenced electronic properties. **(A)** Change in bandgap energy and **(B,C)** band structures computed using mBJ potential.

The calculated results are underestimated in the case of the GGA functional (Iqbal et al., 2023c) and to overcome this underestimation, we have employed another approximation, which is EV-GGA. We have taken advantage of the aforementioned modification based on EV-GGA and compared its results with those of mBJ potential. This comparison showed a similar pattern of the two results in all calculations including the nature of BS, while differing only slightly in the bandwidths of the materials. The bandgap energy of this ternary alloy varies within mBJ in the range of 2.03 eV–2.48 eV and is summarized in Table 2 with the use of different exchange-correlation functionals (see Supplementary Figure S3). The BS analysis shows that the minima of the conduction band and maxima of the valence band are positioned in the same k-vector space, depicting their direct bandgap semiconductor nature (see Figures 2B,C). The material also exhibited the same BS pattern with the GGA, mBJ, and EV-GGA functions, with a slight variation in band-splitting energy. However, despite that variation, the overall BS exhibited the same pattern, so we have displayed them only in the mBJ potential to depict the nature and change in electronic bandgap energy (see Figure 2A) at varying pressures as shown in Figure 2.

**TABLE 2:** Change in electronic bandgap energy of the Cd_0.75_Zn_0.25_Se alloy at varying pressure.

*p* (GPa)	Electronic bandgap (eV)			
	GGA	EV-GGA	mBJ	Literature
0	0.487	1.436	2.025	1.39 (Iqbal et al., 2023a)
				2.27 (Loghina et al., 2019)
				1.00 (Korozlu et al., 2011)
				0.48 (Ameri et al., 2012)
5	0.684	1.665	2.189	----
10	0.815	1.829	2.319	----
15	0.913	1.992	2.418	----
20	1.011	2.091	2.483	----

The incorporation of 75% Cd atoms into the ZnSe unit cell by replacing Zn atoms leads to the formation of impurity bands in the valence band, increasing the degeneracy of the alloy with increasing pressure. At high pressures, these impurity bands shift toward the minimum energy levels, confirming that the bandgap energy of the material is comparatively high. The valence band is noticed to have greater diversity than the conduction band due to its delocalization, leading to an increase in its bandwidth with increasing pressure. This enhancement in bandwidth is not observed in the conduction band. Moreover, when the bandwidth of the valence band is increased, a semiconductor material shows less ionic character, which shows that the ionic character of the Cd_0.75_Zn_0.25_Se alloy decreases with increasing pressure, proving that it is a semiconducting material. With increasing pressure, the valence band electrons shift towards lower values, while the conduction band minima shift toward higher values, showing that the increment of pressure increases the electronic bandgap as the electron energy states are moved to higher energies (see Figure 2). The results computed in the present study are in perfect agreement with the bandgap value of about 2.27 eV at zero pressure obtained in the experimental study (Loghina et al., 2019) in addition to the theoretical results of 1.00 eV (Korozlu et al., 2011) and 0.49 eV and 0.48 eV (Ameri et al., 2012) performed with the use of GGA and LDA functionals. These results motivated us to use improved functionals with better modifications according to the required bandgap in addition to the GGA functional, which would be helpful to further explore the studied material for its effective optoelectronic and photovoltaic applications at different pressure and temperature ranges (Aarifeen and Afaq, 2017; ul Aarifeen and Afaq, 2020; Iqbal et al., 2022b; Iqbal et al., 2023c).

**FIGURE 3:**
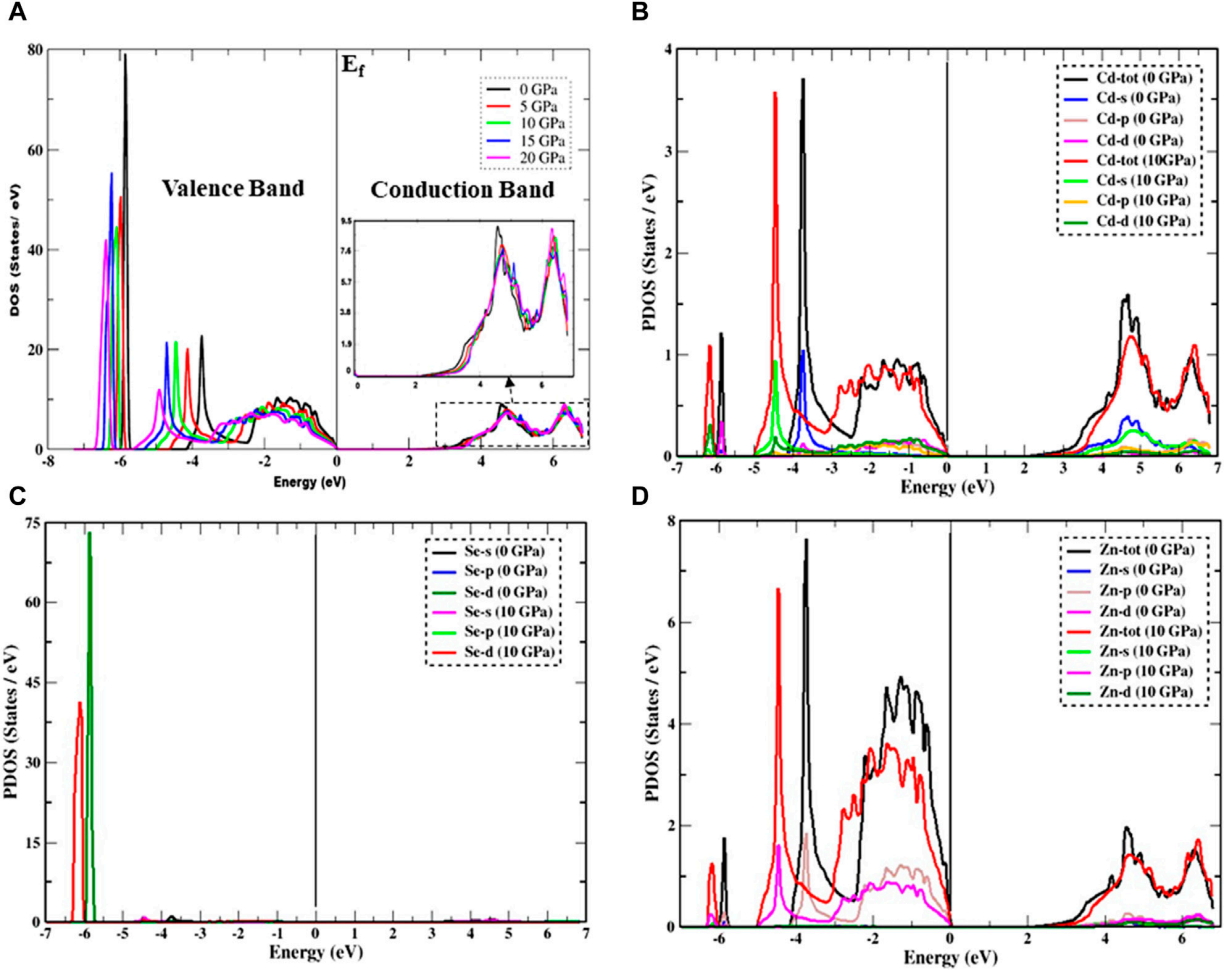
Pressure-impacted variation in DOS. **(A)** TDOS, **(B)** PDOS of the Cd atom, **(C)** PDOS of the Se atom, and **(D)** PDOS of the Zn atom. The inset in **(A)** shows an enlargement of the DOS in the conduction band (right), which enables us to predict the possible changing pattern of the bandgap under the effect of pressure.

The DOS of a material can be used to study the orbital contributions and analyze the nature of the band structure of the material to determine its metallic or semiconducting nature. Both the total (TDOS) and partial density of states (PDOS) are used to gain detailed knowledge of the nature of electronic band structures. The total and partial DOS spectra of the Cd_0.75_Zn_0.25_Se are shown in Figure 3, from which one can acknowledge the semiconducting nature of the material along with the influence of pressure on it. Also, a strong influence of pressure on the DOS of the material can be observed, where such an influence is more prominent in the case of the valence band electrons. We see a peak of DOS at −3.73 eV at zero pressure (the first prominent peak) and this peak shifts to the lower energy of about −4.92 eV (at 20 GPa), while the peak intensity is also noticed to decrease with increasing pressure. In general, it is detected that the intensity of the peaks (the primary peak with higher intensity) at −5.83 eV shifts to −6.38 eV and decreases with increasing pressure, maintaining the overall trend with the only change being in the electronic state density and shifts to low energy radiation. A sharp decrease in peak height is mainly noticed, falling from a higher value of 78.59 units–41.82 units, with the highest peak intensity corresponding to atmospheric pressure (0 GPa). Considering the impact of pressure, the peak intensity declines with increasing pressure, but the general trend of the DOS remains unchanged despite the difference in peak height and the shift to lower energies down to −6.69 eV in the valence band. In general, the electrons shift to lower energy states as the pressure increases, and the peak intensity also drops. On the other hand, with the incorporation of pressure, the DOS produced by electrons in the conduction band is more pronounced without any particular effects. Additionally, under the impact of pressure, the DOS governed by the conduction band electrons has no discernible impact. Figure 3A inset (right) shows the possible trend of bandgap variations with increasing pressure and confirms a significant rise in it. Furthermore, the PDOS of Cd along with the PDOS of Se and Zn atoms at 10 and 0 GPa are shown in Figures 3B–D, which show variations of them for each component in the valence band as Cd-d, s, Se-d, Zn-s, and p states, while only the Cd-p, s, and Zn-p states are in the conduction band. The height of the peak in the conduction band drops with rising pressure and the peak shifts toward low energy levels. These results are in accordance with the reported CdZnSe literature (Iqbal et al., 2022b).

### 3.3 Optical characteristics

For the analysis of the optical characteristics of a material, the light-matter interactions are of particular interest. By studying such interactions, one can learn more about the behavior of the material associated with different interaction phenomena such as transmission, reflection, refraction, and absorption. The interaction of photons (light) with material (matter) induces electronic shifts resulting in an optical spectrum and under various environments, the behavior of the materials is different, which can be determined by studying the complex dielectric function. Depending on their excitation level, incident light excites electrons from an occupied to an unoccupied state, which eventually emits light that has specific wavelengths through common densities. The linear response of a material can be analyzed by using the complex dielectric function, and its derived optical parameters using Equations 10-19, which is mathematically expressed as follows (Fox and Bertsch, 2002):

$$\varepsilon \left(\omega \right)=\varepsilon_{1}\left(\omega \right)+i\varepsilon_{2}\left(\omega \right)$$ (10)

where $\varepsilon_{1}\left(\omega \right)$ is the real and $\varepsilon_{2}\left(\omega \right)$ is the imaginary portion of the dielectric function. The real part provides clear evidence about the polarization using the static dielectric constant at zero frequency, denoted as $\varepsilon_{1}\left(0\right)$ and Kramers–Kronig formulae can be used to compute the real and imaginary portions of the dielectric function using the transformation relations. These formalisms can be given by Johnson, 1975:

$$\varepsilon_{1}\left(\omega \right)=1+\frac{2}{\pi }P\int_{0}^{\infty }\frac{\omega^{/}\;\varepsilon_{2}\left(\omega^{/}\right)}{\omega^{/2}-\omega^{2}}d\omega^{/}$$ (11)

$$\varepsilon_{2}\left(\omega \right)=-\frac{2\omega }{\pi }P\int_{0}^{\infty }\frac{\left(\varepsilon_{1}\left(\omega^{/}\right)-1\right)}{\omega^{/2}-\omega^{2}}d\omega^{/}$$ (12)

where $\omega^{/}$ is the optical angular frequency running through the given integration range, while P denotes the value of Cauchy principal. From the complex dielectric function, as a function of incident light energy, it is possible to infer optical parameters like the refractive index ( $n\left(\omega \right)$ ) and extinction coefficient ( $k\left(\omega \right)$ ). The refractive index depicts the change in speed of light while entering the material, depending on the frequency of incident light, which is presented along with the extinction coefficient, and can be approximated using the relations (Fox and Bertsch, 2002):

$$k\left(\omega \right)=\frac{1}{\sqrt{2}}{\left[\sqrt{\left\{{\varepsilon_{1}}^{2}\left(\omega \right)+{\varepsilon_{2}}^{2}\left(\omega \right)\right\}}-\varepsilon_{1}\left(\omega \right)\right]}^{1/2}$$ (13)

$$n\left(\omega \right)=\frac{1}{\sqrt{2}}{\left[\sqrt{\left\{{\varepsilon_{1}}^{2}\left(\omega \right)+{\varepsilon_{2}}^{2}\left(\omega \right)\right\}}+\varepsilon_{1}\left(\omega \right)\right]}^{1/2}$$ (14)

Furthermore, to analyze the absorption characteristic of the material, the extinction coefficient can be used as a measure of light decay, which affects its penetration inside the material (Fox and Bertsch, 2002). The absorption coefficient can be computed as:

$$\alpha \left(\omega \right)=\frac{4\pi k\left(\omega \right)}{\lambda_{0}}=\frac{\omega }{nc}\varepsilon_{2}\left(\omega \right)$$ (15)

The reflection spectrum can be employed to study the response of the surface of the material (Fox and Bertsch, 2002) and can be expressed as:

$$R\left(\omega \right)=\frac{{\left(n\left(\omega \right)-1\right)}^{2}+k^{2}\left(\omega \right)}{{\left(n\left(\omega \right)+1\right)}^{2}+k^{2}\left(\omega \right)}$$ (16)

In addition, by using the imaginary portion of the dielectric constant, it is possible to examine the optical conductance, a non-contact characteristic of the material (Fox and Bertsch, 2002). The real component of optical conductance can be mathematically written as:

$$Re\sigma \left(\omega \right)=\frac{\omega \;}{4\pi }\varepsilon_{2}\left(\omega \right)$$ (17)

The electron energy loss function of the material can be presented as (Fox and Bertsch, 2002):

$$L\left(\omega \right)=\text{Im}\left(-\frac{1}{\varepsilon \left(\omega \right)}\right)=\frac{\varepsilon_{2}\left(\omega \right)}{\left(\varepsilon_{1}^{2}\left(\omega \right)+\varepsilon_{2}^{2}\left(\omega \right)\right)}$$ (18)

Furthermore, to investigate the connection between the bandgap energy of the material and the real part of the dielectric function (at ω=0 Hz), the Penn model is employed (Penn, 1962) which may be presented as:

$$\varepsilon_{1}\left(0\right)\approx 1+{\left(\frac{\hbar \omega_{p}}{{Ε}_{opt}}\right)}^{2}$$ (19)

Herein, $\hbar \omega_{p}$ represents the plasma energy attributed by plasma frequency $\omega_{p}$ and *E*_opt_ is the optical bandgap energy.

**FIGURE 4:**
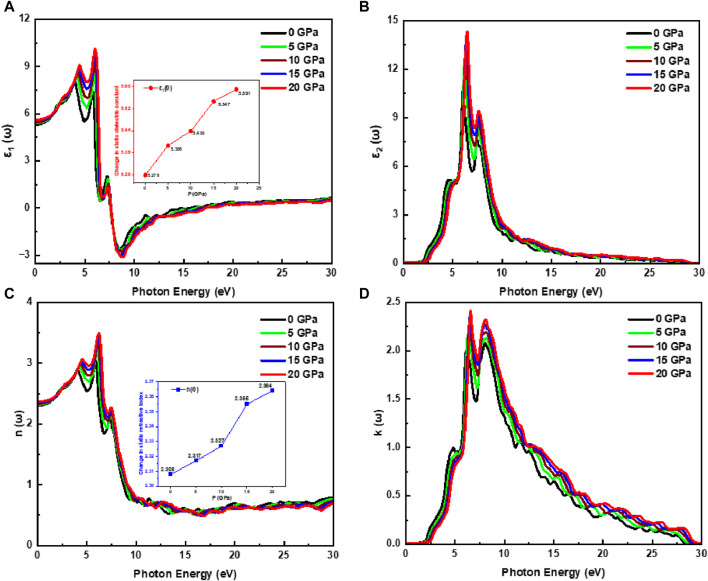
Pressure-influenced change in a dielectric function and refraction. **(A)** $\varepsilon_{1}\left(\omega \right)$ , and **(B)** $\varepsilon_{2}\left(\omega \right)$ , **(C)** refractive index, and **(D)** extinction coefficient. The inset in **(A)** and **(C)** displays the change in $\varepsilon_{1}\left(0\right)$ and $n\left(0\right)$ under the effect of pressure, respectively.

Based on the above, we investigated the optical characteristics of the Cd_0.75_Zn_0.25_Se ternary alloy using mBJ functional with a denser k-points mesh at a pressure ranging from 0 to 20 GPa for an incident light energy of 30 eV. Figure 4 presents the spectrum of the complex dielectric function of the alloy, depicting an increase in its real part followed by a minor decline and then a significant rise to the maximum peak value of 8.02 units at 4.04 eV of incoming radiation (see Figure 4A). This figure depicts the primary electronic energy transitions in a range of 2.22 eV–6.35 eV with a rise in pressure and peak height along with the high values of $\varepsilon_{1}\left(\omega \right)$ . Moreover, the material exhibits metallic characteristics for the incident radiation energies of 7.36 eV–15.86 eV because of the reflection of all of the incoming radiation in this range, confirming the material’s efficacy as a shield for vacuum and ultraviolet radiation in this range of incident radiation. The real part of the dielectric function becomes positive at high-energy incident radiations, for instance, after 17.42 eV, confirming that this high-energy radiation would not affect the material and thus finding applications in optical lenses. However, that shifting tendency of the dielectric constant follows a similar trend with a rise in peak height and a shift toward higher energy radiation with pressure variation. The static dielectric constant is also influenced by pressure variations (see inset in Figure 4A). With the increase in pressure, the electronic bandgap energy of the material increases, and a similar trend is noticed for the static dielectric function. This confirms that the static dielectric constant has the lowest value at the lowest incident radiant energy, which corresponds to the lowest pressure, and *vice versa*. This is mainly because, as the pressure increases, the charge carrier mobility also increases along with a significant increase in dielectric polarization, which finally leads to higher energy eigenvalues. On the other hand, the optical bandgap energy (*E*_opt_) decreases in accordance with the Penn model (see Supplementary Figure S4). However, for the imaginary part of the dielectric function, its threshold energies are associated with the interband transitions of valence band maxima and conduction band minima, as presented in Figure 4B. The imaginary part of the dielectric function experiences a rise in the spectra starting at 1.97 eV, and a higher value of 10.70 units associated with 5.98 eV of incident radiation (at 0 GPa). The transitions from the top of the valence band (Zn-p, Cd-s, and Se-d states) to the bottom of the conduction band occur directly at the critical points (Cd-s, p and Zn-p, d states). The threshold value of 1.97 eV and the peak height of 10.70 units occurred at 5.98 eV for incoming photons when the pressure was 0 GPa, whereas the threshold value was 2.46 eV and the peak height was 14.27 units at 6.38 eV for incoming photons when the pressure was 20 GPa, demonstrating a rise in threshold values as a function of pressure. Three major peaks can be seen in the range between the absorption threshold and 7.85 eV of incoming photons. The threshold value is lowest at atmospheric pressure (0 GPa), and it rises as a result of the pressure influence (0–20 GPa). Moreover, peak height is also influenced by pressure variations, which go on increasing from 10.70 to 14.27 units, while a static spectrum is observed at and above the incident radiation energy of 17.18 eV, presenting a spectral static response. Figure 4C displays the pressure-induced rise in the $n\left(\omega \right)$ with additional peaks observed in the 3.85–7.45 eV range of incoming radiation. A static refractive index ( $n\left(0\right)$ ) plot is revealed in the inset of Figure 4C, which depicts the influence of pressure on static refractive index values that change from 2.308 to 2.364 units with the incident radiation. It shows a static spectrum at and above 32.75 eV, indicating that further increases in incoming radiation do not affect its spectrum. As demonstrated in Figure 4D, the maximum peak height likewise rises with pressure as the extinction coefficient quickly rises as a result of threshold energies. There are no noticeable spectra detected for extinction coefficients over 28.29 eV of radiation because these materials do not interact with photons with energy larger than 28.29 eV. As a result, the absorptance spectrum is unnoticeable in this range. The primary peaks at 0 and 20 GPa pressure are in the 1.98–2.38 unit range, respectively. The index of refraction and extinction coefficient is noticed to be generally rising due to the pressure impact, with the active zone being associated with incoming radiation between 1.73 and 12.44 eV.

**FIGURE 5:**
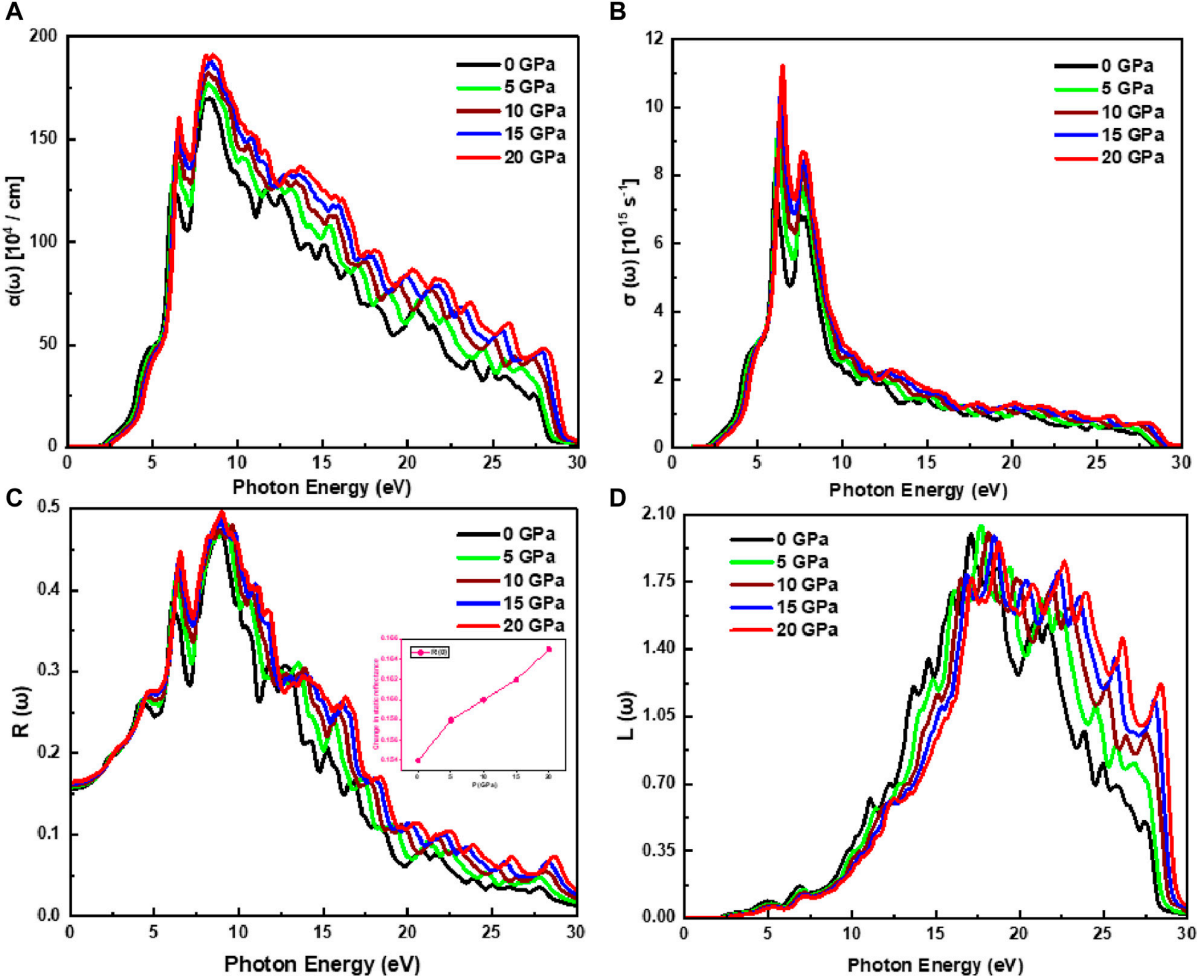
Pressure-influenced change of physical parameters. **(A)** Absorbance, **(B)** optical conductivity, **(C)** reflectance, and **(D)** loss function. The inset in **(C)** shows the variation in $R\left(0\right)$ under the impact of pressure.

Figure 5A shows the absorption spectra of the Cd_0.75_Zn_0.25_Se, which are associated with the imaginary part of the dielectric function and associated with the optical absorbance. Overall, the imaginary components of both the dielectric function and extinction coefficient exhibit a similar trend influenced by pressure variations and can be employed to depict the absorption spectra, which are observed with a minor fluctuation. Furthermore, it is also observed that the ternary alloy retains its stability with pressure variations because the spectral curves are identical with minor shifts arising due to band shifting, regardless of the surface variations. The material’s absorption rises with increasing pressure. For instance, at the maximum pressure of 20 GPa, the absorption peak height increases to 191.97 units from its original value of 169.56 units (at 0 GPa), depicting a maximum optical absorption at the incident photon energy of 8.52 eV. Above 30 eV of incident radiation, a stationary pattern of peaks is observed, but no spectra are observed for energies smaller than the bandgap energy. Moreover, the material shows higher absorption values in the energy range of 6.32 eV–8.52 eV, where the primary peaks appeared due to the transitions of electrons from the valence band to higher energy states, along with the shifting of peaks towards high energy values in the ultraviolet region as a function of applied pressure. For incident energy lower than the bandgap of the material, the optical conductivity of the material is observed to be zero, while this value increases dramatically with rising pressure and presents an inclined peak height of 7.83 units–11.25 units (see Figure 5B). However, in the incident photon energy range from 5.98 eV to 8.75 eV, the new peaks in the spectrum are observed, while at and above the irradiation energy of about 28.47 eV, the spectrum shows a static response with pressure influence, which is in accordance with the extinction coefficient and absorptance spectrum. The Cd_0.75_Zn_0.25_Se alloy exhibits a maximum optical conductivity of 11.25 units at a maximum applied pressure of 20 GPa with an irradiation energy of 6.47 eV, while observing high conductivity values in the incident photon energy range of 5.98–8.75 eV, implying that the material is optically active in this region. Moreover, the spectrum presented in Figure 5B shows the direct influence of pressure on optical conductivity. The increase in pressure excites electrons from the valence band, resulting in the transition of these excited electrons to higher energy band states and, consequently, a tuned optical response that depicts improved optical conductance.

The reflection spectrum presented in Figure 5C depicts a significant rise with increasing pressure and irradiation energy. Also, the peak shifts have been observed. That is, peaks move towards higher values of photon energy as the pressure rises, which are associated with the wide spectra along with the influence on peak height with varying pressure, as reported in the CdZnSe literature (Milstein and Hill, 1979). For the incident light radiation range of 11.13 eV–28.61 eV, the reflection spectrum experiences dispersive peaks and humps. However, the rate of reflection rises with increasing pressure, and the maximum reflectivity of 0.49 units is observed at the maximum pressure value of 20 GPa associated with incoming radiation of 9.01 eV. The static index of reflection also experiences an inclined range from 0.154 units to 0.165 unit depending on pressure variations (0–20 GPa). In addition, at the incoming energy range of 11.13–28.60 eV, new peaks in the spectra are also observed. The optical parameters at zero irradiation frequency along with the optical bandgap energy values are summarized in Supplementary Table S2. However, at lower incident energy values, no electron scattering occurs and no spectra are observed because the function of energy loss is zero, while inelastic electron scattering occurs when the incident radiations are higher than the material’s bandgap energy and the *L* ( ω ) spectra is observed by the irradiation energy. Figure 5D depicts the maximum energy loss function of 2.05 units at 17.17 eV with the maximum pressure value, which faces a decline with decreasing pressure. In the irradiation energy range of 11.05 eV–27.48 eV, the major peaks of the spectrum are observed, wherein the peak height is detected to shift towards a higher electron energy function with increasing pressure. It has also been demonstrated that it is ignorable below threshold levels and over 29.98 eV of incoming radiation. Peak height has been seen to shift toward bigger *L*(ω) values under the action of pressure. It can be inferred that the material’s optical response can be tuned with pressure, and these results support the availability of this alloy as the material for visible-light displays, especially over orange, yellow, green, and cyan.

## 4 Conclusion

In this work, the pressure-induced band nature and optical characteristics of the Cd_0.75_Zn_0.25_Se alloy have been considered using the full-potential linearized augmented plane-wave method within the DFT. This functional alloy exhibits cubic symmetry at all the considered pressure ranges and its bulk modulus rises as a function of pressure. The band structures have been shown and semiconducting nature has been observed. The analysis of the DOS shows that the valence bandwidth increases as pressure rises, leading to an improvement in the covalent character through a decrease in ionicity. The optical properties are also studied, and under the impact of pressure, the locations of all critical points seem to shift toward higher energies. The pattern of the peaks remains identical, apart from the fact that the peak height of the dielectric function (both parts) has increased. It is also observed that optical conductance and absorption increase as pressure increases. Therefore, this alloy can be employed in the manufacture of electronic, optoelectronic, and photonic devices that are functional in the visible orange to the cyan light range at certain pressures at a specific height.

## Data Availability

The original contributions presented in the study are included in the article/Supplementary Material, further inquiries can be directed to the corresponding authors.

## References

[B1] Aarifeen N. U. Afaq A. (2017). Effects of temperature and pressure on thermodynamic properties of Cd_0.25_Zn_0.75_Se alloy. Chin. Phys. B 26 (12), 123103. 10.1088/1674-1056/26/12/123103

[B2] Ameri M. Fodil M. Aoumeur-Benkabou F. Z. Mahdjoub Z. Boufadi F. Bentouaf A. (2012). Physical properties of the Zn_x_Cd_1-x_Se alloys: ab-initio method. Mater. Sci. Appl. 3 (11), 768–778. 10.4236/msa.2012.311112

[B3] Benkabou F. Aourag H. Certier M. (2000). Atomistic study of zinc-blende CdS, CdSe, ZnS, and ZnSe from molecular dynamics. Mater. Chem. Phys. 66 (1), 10–16. 10.1016/s0254-0584(00)00239-x

[B4] Blaha P. Schwarz K. Madsen G. K. Kvasnicka D. Luitz J. wien2k. An augmented plane wave + local orbitals program for calculating crystal properties. 2001;60(1).

[B5] Deo S. R. Singh A. K. Deshmukh L. Paliwal L. J. Singh R. S. Adhikari R. (2014). Structural, morphological and optical studies on chemically deposited nanocrystalline CdZnSe thin films. J. Saudi Chem. Soc. 18 (4), 327–339. 10.1016/j.jscs.2014.01.005

[B6] De Waele S. Lejaeghere K. Sluydts M. Cottenier S. (2016). Error estimates for density-functional theory predictions of surface energy and work function. Phys. Rev. B 94 (23), 235418. 10.1103/physrevb.94.235418

[B7] Engel E. Vosko S. H. (1993). Exact exchange-only potentials and the virial relation as microscopic criteria for generalized gradient approximations. Phys. Rev. B 47 (20), 13164–13174. 10.1103/physrevb.47.13164 10005620

[B8] Fox M. Bertsch G. F. (2002). Optical properties of solids. Am. J. Phys. 70 (12), 1269–1270. 10.1119/1.1691372

[B9] Gul B. Khan M. S. Khan G. Ahmad H. Thounthong P. Khattak S. A. (2022). First-principles calculations to investigate the optoelectronic, and thermoelectric nature of zinc based group II-VI direct band semiconductors. Optik 271, 170143. 10.1016/j.ijleo.2022.170143

[B10] Gupta P. Maiti B. Maity A. B. Chaudhuri S. Pal A. K. (1995). Optical properties of Zn_x_Cd_1−x_Se films. Thin Solid Films 260 (1), 75–85. 10.1016/0040-6090(94)06461-x

[B11] Iqbal M. A. Ashraf N. Shahid W. Afzal D. Idrees F. Ahmad R. (2021). Fundamentals of density functional theory: recent developments, challenges and future horizons. Density Funct. Theory-Recent Adv. New Perspect. Appl.

[B12] Iqbal M. A. Bakhsh S. Ikram M. Sohail M. Islam M. R. Manoharadas S. (2023a). Investigations on the structural and optoelectronic characteristics of cadmium-substituted zinc selenide semiconductors. Front. Chem. 11, 1299013. 10.3389/fchem.2023.1299013 38162394 PMC10754984

[B13] Iqbal M. A. Malik M. Anwar N. Bakhsh S. Javeed S. Maidin S. S. (2023b). Basic concepts, advances and emerging applications of nanophotonics. Arabian J. Chem. 16, 105040. 10.1016/j.arabjc.2023.105040

[B14] Iqbal M. A. Malik M. Bakhsh S. Sohail M. Arellano‐Ramírez I. D. Morsy K. (2023c). Theoretical insights into pressure-driven stability and optoelectronic response of Cd_0.75_Zn_0.25_S alloy for blue–violet display. Adv. Theory Simulations 6 (9), 2300270. 10.1002/adts.202300270

[B15] Iqbal M. A. Malik M. Shahid W. Din S. Z. Anwar N. Ikram M. (2022a). Materials for photovoltaics: overview, generations, recent advancements and future prospects. in Thin Films Photovoltaics.

[B16] Iqbal M. A. Malik M. Shahid W. Irfan S. Alguno A. C. Morsy K. (2022b). Ab-initio study of pressure influenced elastic, mechanical and optoelectronic properties of Cd_0.25_Zn_0.75_Se alloy for space photovoltaics. Sci. Rep. 12 (1), 12978. 10.1038/s41598-022-17218-8 35902680 PMC9334302

[B17] Jin X. Chen W. Li X. Guo H. Li Q. Zhang Z. (2021). Thick-shell CdZnSe/ZnSe/ZnS quantum dots for bright white light-emitting diodes. J. Luminescence 229, 117670. 10.1016/j.jlumin.2020.117670

[B18] Johnson D. W. (1975). A Fourier series method for numerical Kramers-Kronig analysis. J. Phys. A Math. General 8 (4), 490–495. 10.1088/0305-4470/8/4/009

[B19] Kishore N. Nagarajan V. Chandiramouli R. (2019). Mechanical properties and band structure of CdSe and CdTe nanostructures at high pressure-a first-principles study. Process. Appl. Ceram. 13 (2), 124–131. 10.2298/pac1902124k

[B20] Korozlu N. Colakoglu K. Deligoz E. Ciftci Y. O. (2011). The structural, electronic and optical properties of Cd_x_Zn_1−x_Se ternary alloys. Opt. Commun. 284 (7), 1863–1867. 10.1016/j.optcom.2010.11.032

[B21] Krishnan B. Shaji S. Acosta-Enríquez M. C. Acosta-Enríquez E. B. Castillo-Ortega R. Zayas M. E. (2019). Group II–VI semiconductors. Semiconductors: synthesis, properties and applications, 397–464.

[B22] Liu B. Guo Y. Su Q. Zhan Y. Chen Z. Li Y. (2022). Cadmium-doped zinc sulfide shell as a hole injection springboard for red, green, and blue quantum dot light-emitting diodes. Adv. Sci. 9 (15), 2104488. 10.1002/advs.202104488 PMC9131609 35240001

[B23] Loghina L. Chylii M. Kaderavkova A. Slang S. Svec P. Rodriguez Pereira J. (2021). Highly efficient and controllable methodology of the Cd_0.25_Zn_0.75_Se/ZnS core/shell quantum dots synthesis. Nanomaterials 11 (10), 2616. 10.3390/nano11102616 34685059 PMC8538963

[B24] Loghina L. Iakovleva A. Chylii M. Svec P. Houdek J. Slang S. (2019). Synthetic development in Cd–Zn–Se quantum dots chemistry. Opt. Mater. 97, 109385. 10.1016/j.optmat.2019.109385

[B25] Loglio F. Telford A. Salvietti E. Innocenti M. Pezzatini G. Cammelli S. (2008). Ternary Cd_x_Zn_1-x_Se nanocrystals deposited on Ag (111) by ECALE: AFM and EXAFS characterization. Electrochimica Acta 53, 6978–6987. 10.1016/j.electacta.2008.01.046

[B26] Mahmood Q. Yaseen M. Hassan M. Ramay S. M. Mahmood A. (2017). Theoretical investigation of optical properties and band gap engineering for Zn_1−x_TM_x_Te (TM= Fe, Co) alloys by modified Becke–Johnson potential. Chin. Phys. B 26 (8), 087803. 10.1088/1674-1056/26/8/087803

[B27] Margapoti E. Alves F. M. Mahapatra S. Lopez-Richard V. Worschech L. Brunner K. (2012). Paramagnetic shift in thermally annealed Cd_x_Zn_1−x_Se quantum dots. New J. Phys. 14 (4), 043038. 10.1088/1367-2630/14/4/043038

[B28] Milstein F. Hill R. (1979). Divergences among the born and classical stability criteria for cubic crystals under hydrostatic loading. Phys. Rev. Lett. 43 (19), 1411–1413. 10.1103/physrevlett.43.1411

[B29] Murali K. R. Austine A. (2009). Deposition of Cd_x_Zn_1−x_Se films by brush electrodeposition and their characteristics. Chalcogenide Lett. 6 (1), 23–28.

[B30] Nguyen H. T. Tran T. T. Bhatt V. Kumar M. Yun J. H. (2022). Photoluminescence properties of CdSe/ZnS quantum dot donor–acceptor via plasmon coupling of metal nanostructures and application on photovoltaic devices. J. Phys. Chem. Lett. 13 (19), 4394–4401. 10.1021/acs.jpclett.2c00903 35546522

[B31] Penn D. R. (1962). Wave-number-dependent dielectric function of semiconductors. Phys. Rev. 128 (5), 2093–2097. 10.1103/physrev.128.2093

[B32] Pu C. Dai L. Li H. Hu H. Liu K. Yang L. (2019). Pressure-induced phase transitions of ZnSe under different pressure environments. AIP Adv. 9 (2). 10.1063/1.5082209

[B33] Sahli B. Bouafia H. Abidri B. Bouaza A. Akriche A. Hiadsi S. (2016). Study of hydrostatic pressure effect on structural, mechanical, electronic and optical properties of KMgF_3_, K_0.5_Na_0.5_MgF_3_ and NaMgF_3_ cubic fluoro-perovskites via *ab initio* calculations. Int. J. Mod. Phys. B 30 (32), 1650230. 10.1142/s0217979216502301

[B34] Singh D. J. (2010). Electronic structure calculations with the Tran-Blaha modified Becke-Johnson density functional. Phys. Rev. B 82 (20), 205102. 10.1103/physrevb.82.205102

[B35] Sutrave D. S. Shahane G. S. Patil V. B. Deshmukh L. P. (2000). Micro-crystallographic and optical studies on Cd_1−x_Zn_x_Se thin films. Mater. Chem. Phys. 65 (3), 298–305. 10.1016/s0254-0584(00)00240-6

[B36] ul Aarifeen N. Afaq A. (2020). Lattice thermal conductivity of Cd_x_Zn_1−x_X (X= O, S, Se, Te) from first principles. Mater. Chem. Phys. 251, 123099. 10.1016/j.matchemphys.2020.123099

[B37] Yang T. Zhu X. Ji J. Wang J. (2020). First-principles study of phase transition, elastic and thermodynamic properties of ZnSe at high pressure. Sci. Rep. 10 (1), 3265. 10.1038/s41598-020-59687-9 32094411 PMC7039993

[B38] Zhang Q. Nie C. Chang C. Guo C. Jin X. Qin Y. (2017). Highly luminescent red emitting CdZnSe/ZnSe quantum dots synthesis and application for quantum dot light emitting diodes. Opt. Mater. Express 7 (11), 3875–3884. 10.1364/ome.7.003875

[B39] Zhang T. Zhang X. Yang P. Bai J. Chang C. Jin X. (2019). Bright alloy CdZnSe/ZnSe QDs with nonquenching photoluminescence at high temperature and their application to light-emitting diodes. J. Nanomater. 5, 1–8. 10.1155/2019/9257018

