# Supplementary material for: Pressure induced mechanical, elastic, and optoelectronic characteristics of Cd0.75Zn0.25Se alloy
Source: Front Chem. 2024 Aug 1;12:1405315. doi: 10.3389/fchem.2024.1405315 (PMC11325222; doi:10.3389/fchem.2024.1405315)
Supplement: Supplementary file 1 [file DataSheet1.docx]

**Pressure Induced Mechanical, Elastic, and Optoelectronic Characteristics of Cd_0.75_Zn_0.25_Se Alloy**

Muhammad Aamir Iqbal^1,^*, Saher Javeed^2^, Sunila Bakhsh^3^,  Iván D. Arellano-Ramírez^4^, Muhammad Khalid^3^, Kareem Morsy^5^, Ali A. Shati^5^, Jeong Ryeol Choi^6,^*

1. School of Materials Science and Engineering, Zhejiang University, Hangzhou, 310027, China
2. Department of Physics, Government College University Lahore, Lahore 54000, Pakistan
3. Department of Physics, Balochistan University of Information Technology, Engineering and Management Sciences, Quetta 87300, Pakistan
4. Department of Physics, Universidad Tecnológica de Pereira, Pereira 660003, Colombia
5. Biology Department, College of Science, King Khalid University, Abha 61421, Saudi Arabia
6. School of Electronic Engineering, Kyonggi University, Suwon, Gyeonggi-do 16227, Republic of Korea

***Corresponding Authors Email:** [aamir.hum@gmail.com](mailto:aamir.hum@gmail.com) (MA Iqbal); [choiardor@hanmail.net](mailto:choiardor@hanmail.net) (JR Choi)


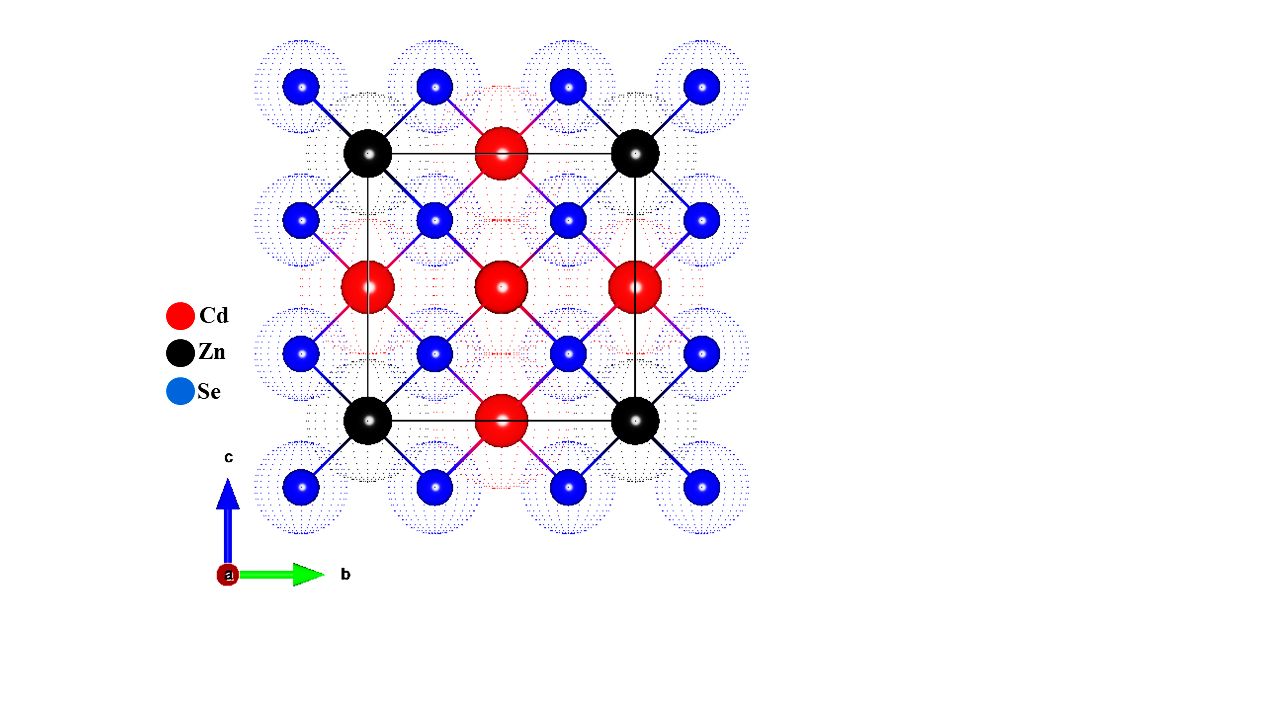


**Figure S1:** Cubic phase crystal structure of the Cd_0.75_Zn_0.25_Se alloy along a axis with a dot surface (viewed in VESTA).


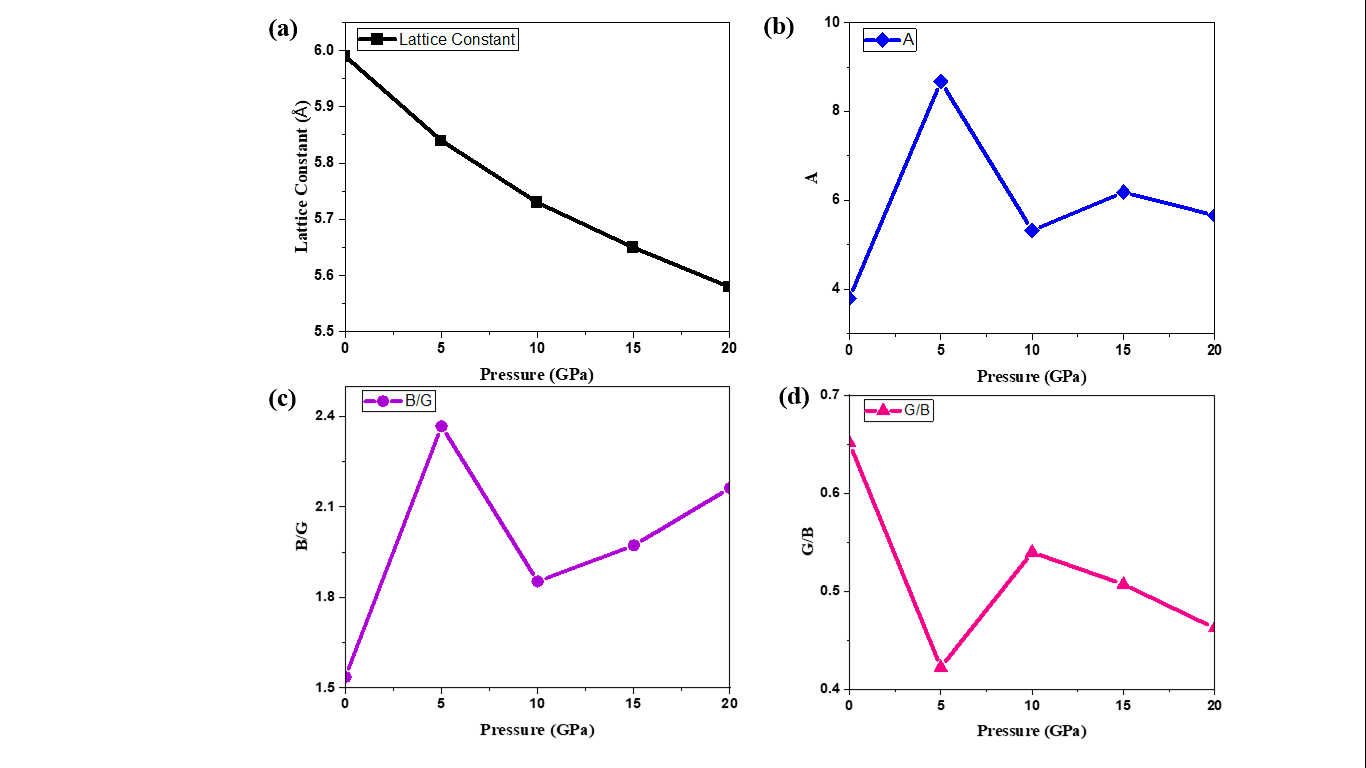


**Figure S2:** Pressure-impacted variation in parameters. **(a)** lattice constant, **(b)** anisotropic factor, **(c)** B/G ratio, and **(d)** G/B ratio.

**Table S1:** The elastic moduli C_11_, C_12_, C_44_ (GPa), lattice constant a (Å), bulk modulus (GPa), and several factors needed for estimating the mechanical stability of Cd_0.75_Zn_0.25_Se alloy depending on the pressure P (GPa).

| P | a | B | C_11_ | C_12_ | C_44_ | C_11_-C_12_ | C_11_ + 2C_12_ | C_11_+C_12_+P | C_11_-C_12_-2P |
| --- | --- | --- | --- | --- | --- | --- | --- | --- | --- |
| 0 | 5.99 | 56.85 | 78.94 | 45.8 | 62.86 | 33.14 | 170.54 | 124.74 | 33.14 |
| 5 | 5.84 | 76.67 | 87.83 | 71.09 | 72.64 | 16.74 | 230.01 | 163.92 | 6.74 |
| 10 | 5.74 | 95.93 | 120.83 | 83.47 | 99.43 | 37.36 | 287.77 | 214.3 | 17.36 |
| 15 | 5.65 | 114.41 | 139.68 | 101.77 | 117.18 | 37.91 | 343.22 | 256.45 | 7.91 |
| 20 | 5.58 | 130.89 | 158.83 | 116.92 | 118.77 | 41.91 | 392.67 | 295.75 | 1.91 |
| 25 | 5.53 | 145.59 | 153.09 | 141.84 | 137.84 | 11.25 | 436.77 | 319.93 | -38.75 |

**Figure S3:** Pressure-influenced change in electronic bandgap values of the Cd_0.75_Zn_0.25_Se alloy computed by employing different functionals.

**Table S2:** Optical parameters at an irradiation frequency of zero.

| P (GPa) | $\boldsymbol{\varepsilon}_{\boldsymbol{1}}\left( \boldsymbol{0} \right)$ | $\boldsymbol{n}\left( \boldsymbol{0} \right)$ | $\boldsymbol{R}\left( \boldsymbol{0} \right)$ | *E*_opt_ (eV) |
| --- | --- | --- | --- | --- |
| 0 | 5.278 | 2.308 | 0.154 | 2.35 |
| 5 | 5.386 | 2.317 | 0.158 | 2.29 |
| 10 | 5.439 | 2.327 | 0.160 | 2.25 |
| 15 | 5.547 | 2.355 | 0.162 | 2.19 |
| 20 | 5.591 | 2.364 | 0.165 | 2.16 |

**Figure S4:** Pressure-influenced change in static dielectric constant and optical bandgap energy.
